# A Systematic Review of the Randomized Controlled Trials of Gemcabene and Its Therapeutic Uses

**DOI:** 10.7759/cureus.36811

**Published:** 2023-03-28

**Authors:** Smruti M Besekar, Sangita D Jogdand, Waqar M Naqvi

**Affiliations:** 1 Pharmacology, Jawaharlal Nehru Medical College, Datta Meghe Institute of Medical Sciences, Wardha, IND; 2 Research, Humen Edutech, Nagpur, IND; 3 Pharmacology and Therapeutics, Datta Meghe Institute of Higher Education & Research, Wardha, IND; 4 Department of Physiotherapy, Gulf Medical University, Ajman, ARE

**Keywords:** review, randomised controlled trial, statins, lipid lowering, gemcabene

## Abstract

Statins are the most widely used drugs for reducing lipid and cholesterol levels in the blood. However, statins have shown some adverse effects and less acceptance among patients; hence, new drugs have been promoted in the market. Furthermore, gemcabenes were discovered in 1995 and are now in phase II and III clinical trials. Gemcabene acts by inhibiting incorporation of 14C-acetate into hepatocytes and stops the mechanism of fatty acids and cholesterol synthesis. In this review the six randomized controlled trials (RCTs) were scrutinized from the two databases by using keywords "Gemcabene" AND "Randomized Controlled Trial.” The trials were mainly on animal models, and two studies were found to be associated with human subjects. The study concluded that gemcabene was effective as an anti-inflammatory agent and reduced lipid levels and the progression of fibrosis. Hence, further controlled trials are needed to determine its efficacy and safety in human subjects, along with the identification of adverse effects.

## Introduction and background

Gemcabene is considered a new pharmaceutical agent that reduces hepatic apolipoprotein C-III (apoC-III) messenger RNA and increases the removal of very-low-density lipoprotein (VLDL). Gemcabene is given as 6, 6'-oxybis [2, 2- dimethyl-4-hexanoic acid] mono-calcium salt and may be prescribed to patients who are incapable of acquiring a normal range of low-density lipoprotein cholesterol (LDL-C) or triglycerides with currently accepted management, especially statin therapy. Gemcabene inhibits the incorporation of 14C-acetate into hepatocytes, which prevents the biosynthesis of both fatty acids and cholesterol [1].

In general, this drug acts by lowering LDL-C and triglycerides and elevating high-density lipoprotein cholesterol (HDL-C) [2,3]. The Food and Drug Administration (FDA) required two-year rat and mouse carcinogenicity tests to be completed, and the results were submitted in January 2016, which led the FDA to partially suspend gemcabene phase II clinical research. The FDA received the required data in May 2018; nevertheless, it judged that the data were insufficient at that time to release the partial clinical hold. Hence, in 2020, the hold was removed, and the gemcabene trial was started again [2]. 

This systematic review was performed to assess different trials from 2017 to 2022 on the use of gemcabenes in different settings, and its future scope.

## Review

Research methodology

**Figure 1:**
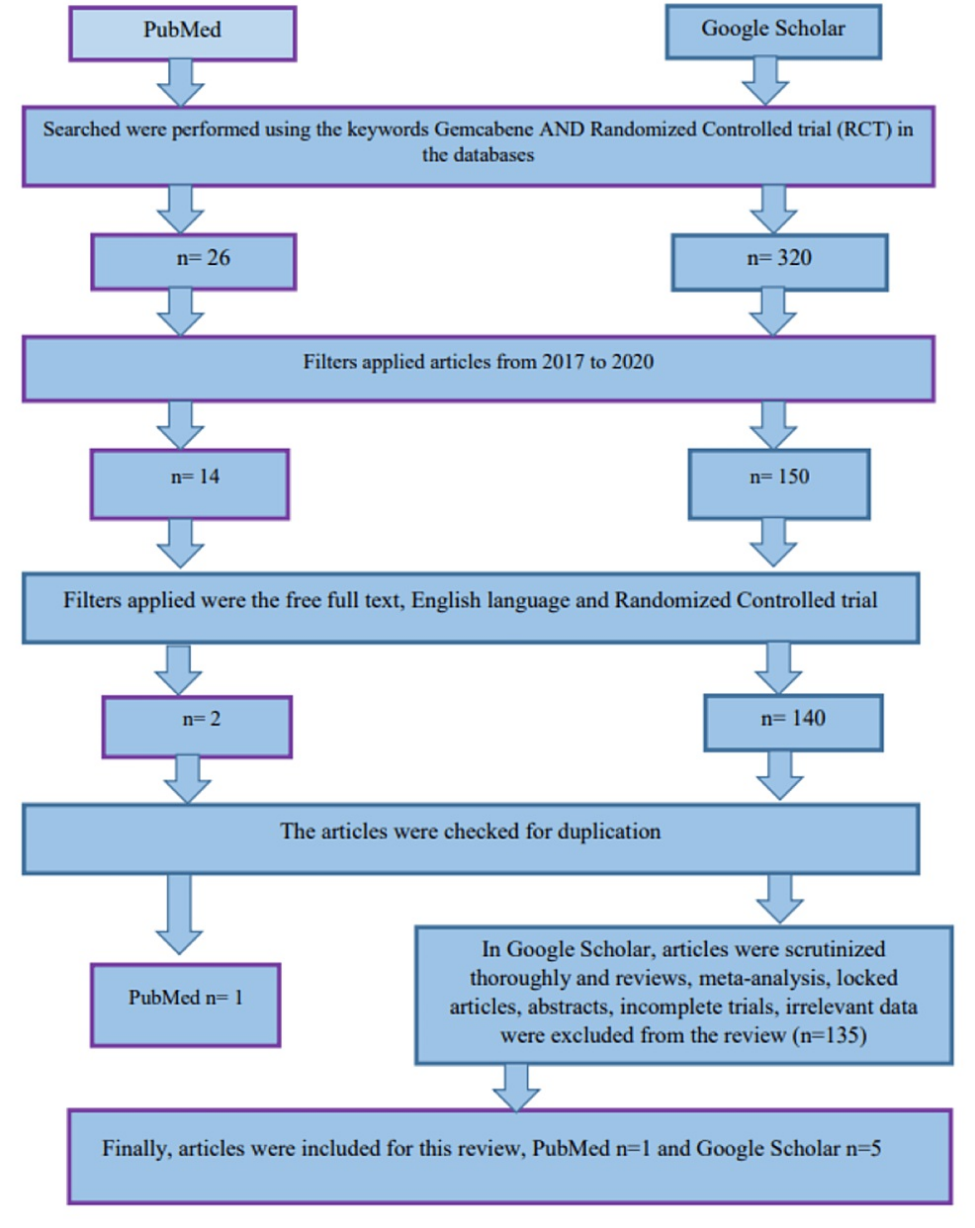
The search strategy were performed by using keywords Gemcabene and Randomized controlled trials.

The systematic review was conducted according to the updated Preferred Reporting Items for Systematic review and Meta-Analyses (PRISMA) guidelines [4]. The search strategy involved randomized controlled trials (RCTs) that were thoroughly reviewed to determine the effectiveness of gemcabenes in different studies. Online searches for articles published between 2017 and 2022 were conducted using Google Scholar and PubMed. The search was conducted using the Boolean operator "AND" between the two keywords Gemcabene AND Randomized controlled trials. The inclusion criteria were as follows: title word, free full-text, randomized controlled experiment, and English language. Articles that were part of systematic reviews, meta-analyses, original articles, citations, patents, incomplete clinical trials, abstracts, locked articles, or studies conducted before 2017 were excluded. Figure 1 shows the search strategy for the databases.

Results

Six RCTs were enrolled from a total of 346 articles that included gemcabenes. From these RCTs the majority were pre-clinical and conducted on animal models, and only two trials were conducted on human patients. Table 1 shows the RCTs that were enrolled in this systematic review and their information, such as author name, place of the trial, database, and publication date. Table 2 depicts the study’s type of participants, methods, objectives, and conclusions.

**Table 1:** Information about the randomized controlled trials involved in the review.

Sr No	Name of the Author	Database	Place of the study	Publication date
1	Srivastava K et al. [5]	Google Scholar	United States	2018
2	Gaudet et al. [6]	PubMed	United States, Canada, Isreal	2019
3	Oniciu et al. [7]	Google Scholar	Japan	2018
4	Srivastava K et al. [2]	Google Scholar	United States	2018
5	Bisgaier et al. [8]	Google Scholar	United States Of America	2018
6	Stein et al. [9]	Google Scholar	Cincinnati	2017

**Table 2:** The data about the randomized controlled trials' inclusion, purpose, method and outcome. NASH- Nonalcoholic steatohepatitis, CRP- C-reactive protein, PPAR- Peroxisome proliferator-activate receptor, NAS- NAFLD Activity Score, LDL-C- Low-density lipoprotein- cholesterol, EBP- Enhancer binding protein, NF-B- Nuclear factor kappa beta, IL- Interleukin, STAM™ murine model- male mouse model, QD- quaque die (daily administration).

Sr No	Authors name	Inclusion	Purpose	Method	Conclusion
1	Srivastava et al. [5]	Sprague Dawley rats ( Male and 200–300 g)	Evaluated the anti-inflammatory characteristics of gemcabene, its anti-inflammatory activities.	Gemcabene was dissolved in 0.05% hydroxypropyl methylcellulose or 0.2% Tween 80 vehicle and then applied to the joint and cartilage structures using two different dosage paradigms.	The study showed that gemcabene found as an anti-inflammatory agent and abates inflammatory disease in rats.
2	Gaudet et al. [6]	Eight Patients with hypercholesterolemia patients.	The effectiveness, desirability, and safety of gemcabene as an additional therapy to standard lipid reducing therapy for patients with familial hypercholesterolemia were examined in the (COBALT-1) study.	Patients was administered with 300 mg gemcabene for the first month, 600 mg for the next second month, and 900 mg for the final last month.	According to COBALT-1 research, gemcabene has the capability to considerably lower LDL-C range in patients with familial hypercholesterolemia when used in union with existing lipid reducing drugs.
3	Oniciu et al. [7]	2 day old neonatal C57BL/6 male mice	“Based on the combination of lipid-lowering and anti-inflammatory efficacy”in clinical trials, and was evaluated for its potential value in a preclinical subjects of NASH (Non-alcoholic steatohepatitis)	STAM™ murine model of NASH was used to test gemcabenes. In mice fed a high-fat, high-calorie diet after the induction of diabetes with streptozotocin, gemcabene treatment was evaluated for changes in plasma, liver histology, and mRNA indicators of lipid metabolism and inflammation. murine model of NASH. Gemcabene.	The study suggested that gemcabene may be a good candidate for reducing the progression of both NAS and fibrosis based on histological findings in gemcabene-treated STAM™ mice.
4	Srivastava et al. [2]	Human hepatoma cell monolayers.	To comprehended the system of CRP ( C-reactive protein) reduction, an investigation was conducted	Transfection investigations using human CRP regulatory course in luciferase/-gal systems were performed.	Gemcabene inhibits IL-6- and IL-1-induced CRP synthesis and lowers CRP through a transcriptional mechanism mediated by C/EBP and NF-B.
5	Bisgaier et al. [8]	Chinese hamster ovary, mouse, rat, and human PPAR (peroxisome proliferator-activate receptor) subtype constructs.	Transactivation experiments were used to examine the possible contribution of PPAR subtype activity to PPAR subtype constructs.	Using a proprietary cell line, assays were carried out in 96-well plates	These findings indicate that Gemcabene is ineffective as a direct agonist or antagonist of all three PPAR subtypes :-α, γ, and δ, in humans, rats, and mice. They further suggested that gemcabene did not significantly bind to or directly activate PPAR nuclear hormone receptors
6	Stein et al. [9]	The study included men and postmenopausal women 18 to 65 years old and LDL-C (130 mg/dL (3.4 mmol/L	Evaluated the safety and effectiveness of gemcabene when used in addition to steady statin therapy in hypercholesterolemic individuals.	The study were 2 month, randomized, phase 2 research iconducted on males and postmenopausal women between the ages of 18 and 65 years who were using stable, low-to-moderate-intensity statin therapy (the majority were taking low-intensity statins). Gemcabene 300 mg, gemcabene 900 mg, or placebo QD were randomly assigned to sixty-six participants in a 1:1:1 ratio.	In comparison with placebo, gemcabene showed additional dose-dependent and statistically significant reductions in LDL-C (> 20 %) and CRP (> 40 %). These findings support the further development of gemcabenes for individuals who need LDL-C lowering over and beyond what is offered by routine statin therapy.

Discussion

For the treatment of osteoarthritis, hypertension, familial partial lipodystrophy (FPL) disease, homozygous familial hypercholesterolemia (HoFH), heterozygous familial hypercholesterolemia (HeFH), non-alcoholic steatohepatitis (NASH), non-alcoholic fatty liver disease, and non-familial hypercholesterolemia in atherosclerotic cardiovascular disease (ASCVD) patients, gemcabene calcium is currently being researched for this diseases [10]. Additionally, it was developed to treat mixed dyslipidemia and hypertriglyceridemia and is also being developed for acute COVID-19 caused by severe acute respiratory syndrome coronavirus-2 (SAR-Cov-2). It is also used for familial chylomicronemia syndrome (FCS), Alzheimer’s disease, tauopathies, and pancreatitis [10-13].

Low low-density lipoprotein cholesterol (LDL-C) levels have been considered an efficient and controllable risk factor for lowering the occurrence of cardiovascular events (CVD) during the past three decades, and many large prospective clinical trials have been conducted on this key mechanism. More efficient and tolerable pharmacological methods to lower LDL-C levels are required because the majority of alternative lipid-modifying treatments currently in use have inadequate efficacy or tolerability [14,15]. Dyslipidemia plays a vital role in the development of cardiovascular disease, and various new therapies for lipid reduction have been developed. Inclisiran has recently been developed as an RNA targeting the hepatic proprotein convertase subtilisin/kexin type 9 (PCSK9) inhibitor, which, when combined with other lipid-lowering medications, accelerates LDL receptor recycling and potently reduces LDL cholesterol by 51% [16,17]. Evinacumab is a monoclonal antibody that inhibits angiopoitein-like protein-3 (ANGPTL3), reduces LDL cholesterol regardless of LDL receptor function, and is a potentially useful drug [18]. Studies on vupanorsen, which uses an antisense oligonucleotide to target ANGPTL3, also revealed statistically significant dose-dependent decreases in LDL cholesterol, triglycerides, non-HDL cholesterol (non-high-density lipoprotein), and total cholesterol [19]. ARO-ANG3, a small interfering RNA (siRNA) directed against ANGPTL3 messenger RNA (mRNA) currently in phase II clinical research, and bempedoic acid, which inhibits ATP-citrate lyase, are two additional therapeutic options that effectively lower LDL cholesterol levels [16,20,21].

Statins play a crucial role in LDL-C reduction, but some patient populations cannot tolerate these drugs, and have experienced contraindications, or unable to reach consensus-based "ideal" or "target" LDL-C values despite having a sufficient response to statins and other LDL-C controlling medications [22-24].

Many LDL-C-reducing medications approved by the FDA, such as proprotein convertase subtilisin/kexin type 9 (PCSK9) inhibitors, single-chain antibodies (alirocumab and evolocumab), niacin-related substances, peroxisome proliferator activated receptor (PPAR) agonists, acetyl Co-A carboxylase (ACC) inhibitors (gemcabene), adenosine triphosphate-citrate lyase, adenosine monophosphate-activated protein kinase modulators (bempedoic acid), and niacin-related substances, are now available in the market [10].

In 1995, a number of carboxy alkyl ethers, including gemcabenes, were registered. The former preclinical outcomes of gemcabene (PD72953) and a few successive drugs were published in 1998 [10]. In 2011, Michigan Life Therapeutics (Ann Arbor, MI, USA) obtained a license for the gemcabene program from Pfizer Inc., and a new investigational new drug (IND) proposal for gemcabenes was submitted in 2015. Gemcabene has been dispensed to 895 patients and healthy volunteers for up to 12 weeks and has been found to be tolerable up to 900 mg [25,26]. Gemcabene treatment reduced joint swelling, and lessened paw withdrawal latency in the carrageenan-induced thermal hyperalgesia (CITH) rat model of osteoarthritis (OA). These results were further supported by the interleukin IL-6 and 6sR knee injection paradigms in rats, which demonstrated significant reductions in the weight distribution of the hind paws at doses of 10 mg and 30 mg, respectively. Gemcabene was found to be effective as an anti-inflammatory agent in animal models of inflammation-induced arthritis and hyperalgesia [8].

Another study involved patients with HoFH, which is an atypical hereditary condition characterized by early ASCVD and significantly high plasma LDL-C. Most patients with HoFH respond moderately to statins, ezetimibe, and proprotein convertase subtilisin/kexin type 9 (PCSK9) inhibitors depending on LDL receptor (LDLR) activity [27]. In the COBALT-1 study, eight patients were enrolled in the gemcitabine treatment group, and the dose was increased from 300 to 900 mg once daily without a break in dosing. The study's 18-week duration and atherogenic lipid and lipoprotein levels (non-HDL-C, ApoB, and ApoE) were significantly reduced by 30%, comparable to the results seen with statins, ezetimibe, and PCSK9 inhibitors; no changes were observed in other lipid parameters, such as HDL-C, LDL-C, ApoAI, ApoAII, ApoCIII (apolipoprotein), and triglycerides (TG). Although side effects, such as headaches, diarrhea, and treatment-related adverse events have been reported, no fatalities or significant life-threatening incidents have been experienced. The levels of liver enzymes remained unchanged, although serum creatinine levels increased slightly [6].

Similarly, untreated liver fat deposition may lead to non-alcoholic fatty liver disease (NAFLD) and its deadly variant, NASH, which causes fibrosis, liver failure, cirrhosis, and hepatocellular carcinoma (HCC). As such, there is no acceptable drug therapy exists for the treatment of NASH, but drugs such as vitamin E and pioglitazone, although not approved for NASH, have demonstrated a significant reduction in hepatic steatosis and inflammation, but cannot slow down the progression of NAFLD. To identify viable clinical therapeutic candidates for NASH, translational medicine combines detection methods, disease markers, and cutting-edge therapies, many of which have already been established for dyslipidemia [28]. In comparison with the NASH group treated with the vehicle, gemcabene also dramatically decreased (low and high doses) or had a propensity to mid-dose. Gemcabene- and telmisartan-treated mice displayed less lobular swelling and inflammation than vehicle-treated NASH mice, and NASH was significantly decreased compared with vehicle-treated NASH mice. Although it gradually decreased, there was no discernible difference in NAFLD Activity Score (NAS) between NASH mice treated with vehicle (100 mg/kg) and gemcabene (100 mg/kg). Nonetheless, gemcabene significantly reduces hepatic mRNA markers of inflammation, lipogenesis, and lipid modulation [7].

The path of atherosclerosis is significantly stimulated via way of means of inflammation, and the high-sensitivity C-reactive protein (CRP) level has been recognized as a predictor of cardiovascular risk. Gemcabene, both alone and in combination with statins, significantly decreased CRP levels in humans. Additionally, gemcabene may indirectly lower plasma CRP levels by lowering LDL levels, which raises the possibility that gemcabene inhibits the action of proinflammatory cytokines in the liver via a transcriptional mechanism. This could account for the success of this drug in lowering CRP levels in clinical trials [8].

The ability of gemcabene to reduce IL-1-induced inflammation and CRP production in this trial and the ability of canakinumab to reduce CVD events in the CANTOS study imply that anti-inflammatory drugs may reduce CVD events. Therefore, the anti-inflammatory properties of gemcabene provide patients with CVD with additional benefits in addition to its LDL-lowering action [2,18].

PPARs are ligand-activated transcription factors that belong to the nuclear receptor superfamily. PPARs are type II nuclear receptors that have a DNA-binding domain with a cysteine-rich zinc-finger motif. PPAR is a metabolic pathway regulator in CVD, and gemcabene showed almost minimal PPAR activity against rats and mice in the assay settings and constructs used, but low marginal PPAR activity against humans at the highest concentration (300 µM) examined. Rosiglitazone, a known PPAR activator, demonstrated significant agonist activity, as expected, whereas gemcabene showed minimal activation at 300 µM and no activity at 100 µM, indicating that gemcabene may or may not directly activate PPARs depending on the species. Gemcabene does not appear to have a distinct antagonist activity against any PPAR subtype (human, mouse, or rat), according to the findings, and there is an obvious absence of concentration-response [9]. In this study, gemcabene, which is highly compatible and has a long-term safety comparable to that of statin medication alone, was administered to individuals following stable statin therapy. The results of this study support the continued development of gemcabene as an alternative medication to lower LDL-C levels in patients with CVD who are unable to achieve their goals with their current statin therapy, and in patients without CVD who have severe hypercholesterolemia, including HoFH or HeFH. The study was conducted on a small number of patients; therefore, the certainty about the TG levels was vague, as adjuvant therapy with statins was administered; hence, the efficacy and safety of gemcabene was not assessed efficiently, and the tested gemcabene was administered to patients with an LDL-C level of 130 mg/dl [10]. In the INDIGO-1 study, gemcabene 600 mg was found to be beneficial in reducing triglyceride levels in patients with severe hypertriglyceridemia [29,30]. In pediatric NAFLD, patients were focused on the development of a phase 2a proof-of-concept (POC) to investigate gemcabene as a treatment option [31].

The weakness of this systematic review was the small number of RCTs included; hence, a comprehensive conclusion could not be reached. A small number of trials were conducted with human subjects; therefore, their efficacy and safety were not evaluated.

In the near future, extensive research should be conducted on the treatment of these conditions after regulatory approval. The gemcabene market is expected to change in the coming years. Increased healthcare costs will proprotionally increase market size and allow drug manufacturers to expand their market share. Large pharmaceutical companies and researchers are analyzing problems and looking for opportunities that might affect gemcabene supremacy. The focus of currently developed therapies is on cutting-edge methods to treat or ameliorate the disease state [2,32]. 

## Conclusions

In this review, gemcabene was found to be an effective anti-inflammatory agent that potentially decreased LDL-C levels alone or when prescribed with other lipid-lowering agents, such as statins, in the medical care of patients with familial hypercholesterolemia or in postmenopausal women. It also showed a significant reduction in fibrosis progression in NASH patients and decreased levels of CRP, IL-6 and IL-1 beta. Hence, most studies have been conducted on animal models, and their efficacy and safety in humans have not been fully investigated. Therefore, further studies are needed to evaluate gemcabenes in human clinical settings.
